# The method described by Czosnyka is particularly suitable for measuring CPPe in patients undergoing cerebral angiography

**DOI:** 10.3389/fsurg.2024.1488265

**Published:** 2025-01-06

**Authors:** Yunyun Liang, Pei Mo, Yonghong Chen, Xinwu Liu, Lin Chen, Xiaomin Zhou, Zijing Wang, Junyi Fu, Longchang Xie

**Affiliations:** ^1^ Department of Rehabilitation Medicine, The Second Affiliated Hospital, Guangzhou Medical University, Guangzhou, China; ^2^ Department of Cardiology, The Second Affiliated Hospital, Guangzhou Medical University, Guangzhou, China; ^3^ Department of Neurology, Institute of Neuroscience, Key Laboratory of Neurogenetics and Channelopathies of Guangdong Province and the Ministry of Education of China, The Second Affiliated Hospital, Guangzhou Medical University, Guangzhou, China; ^4^ College of Clinical Medicine, Guilin Medical University, Guilin, China

**Keywords:** cerebral perfusion pressure, cerebral blood flow, resistance-area product, cerebrovascular resistance, critical closing pressure

## Abstract

**Background:**

The primary objective of this study was to estimate the effective cerebral perfusion pressure (CPPe), critical closing pressure (CrCP), and resistance-area product (RAP) of the intravascular common carotid artery using three different methods. These estimates were then compared to the reference method of linear regression (LR).

**Methods:**

In our previous study, we employed linear regression to evaluate the values of CrCP and RAP. To assess the consistency of results obtained from alternative assessment methods (CPPe, CrCP, and RAP) with the linear regression LR, we conducted a secondary analysis of the previously collected data. We estimated the CPPe, CrCP, and RAP of the intravascular common carotid artery using three different methods: Belford's method (mean/diastolic pressure), Czosnyka's method (systolic/diastolic pressure, CZO), and Schmidt's method (systolic/diastolic pressure, SCH), and compared these estimates with LR. CPPe is calculated as the difference between mean arterial pressure and CrCP. The primary outcome was the mean differences and biases between CPPe, CrCP, and RAP of intravascular common carotid artery, the secondary outcome was correlations and agreement among these various estimates of CPPe measurements.

**Results:**

Nineteen patients were included in this analysis. The median age was 53.5 ± 11.6 years, with 73.7% being men. There were no significant differences in CPPe, RAP and CrCP between the right common carotid artery (RCCA) and the left common carotid artery (LCCA) by using three different methods. Compared to the LR, the mean differences in CPPe and CrCP values were no significant for LCCA according to SCH, CZO and BEL method. But for RAP, the three methods are different in terms of mean differences compared with the LR. CPPe and CrCP revealed a small mean bias compared CPP_CZO_ with CPP_LR_. Comparing CPPLR measurements with CPPBEL, the mean bias was higher with wider LoA. BEL and CZO showed a strong correlation with LR in Pearson correlation coefficients.

**Conclusion:**

The CPPe, CrCP, and RAP values obtained using the CZO calculation methods are comparable to those measured using the reference method. These findings may provide valuable insights for patients undergoing digital subtraction brain angiography, aiding in the determination of the most suitable approach for individualized blood pressure management.

## 1 Introduction

The maintenance of constant cerebral blood flow (CBF) is essential for normal brain function (1). Among the various mechanisms that regulate CBF, cerebral autoregulation (CA) is recognized as one of the most critical physiological processes. Cerebral autoregulation involves the adjustment of cerebral blood vessel tone in response to fluctuations in cerebral perfusion pressure (CPP), thereby ensuring stable CBF. Under physiological conditions, CA can be assessed clinically by measuring CBF or other substitutes related to CPP. When CA is intact and the mean arterial pressure (MAP) falls within the CA range, variations in CPP do not affect CBF. Conversely, when CA is impaired or MAP is outside the CA range, changes in CBF become directly dependent on CPP. Maintaining CPP above a certain threshold can mitigate cerebral ischemia; however, excessive CPP may lead to cerebral edema or induce systemic complications (2). CPP serves as a major determinant of CBF, and its monitoring is fundamental in the practice of neurosurgery (3).

Dewey et al. indicated that the cerebral perfusion pressure (CPPe) of cerebral autoregulation (CA) can be calculated by measuring the critical closing pressure (CrCP). Dewey posited that within the effective regulatory range of CA, a decrease in blood pressure corresponds to a reduction in the contraction of vascular smooth muscle, leading to a decline in cerebrovascular tone and CrCP. Ultimately, when the smooth muscle is fully relaxed, CrCP reaches zero and does not decrease further with additional reductions in blood pressure. At this juncture, the effective perfusion pressure can no longer remain constant and begins to fluctuate in response to changes in blood pressure, resulting in a decrease in cerebral blood flow (4). The mean arterial pressure (MAP) at this point is equivalent to CPPe, which can be simply calculated by subtracting CrCP from MAP prior to the change in blood pressure (5).

Since the introduction of transcranial Doppler sonography, several methods have been developed to assess brain CrCP, CPPe, and resistance-area product (RAP) through the evaluation of pressure-velocity relationships (6–8). CrCP is the pressure at which CBF reaches zero, and it can be extrapolated through line regression (LR) of CBFv-ABP. Meanwhile, the RAP refers to the slope of the instantaneous pressure-velocity relationship, calculated indirectly by dividing the mean cross-sectional blood flow velocity by the absolute flow divided by the arterial cross-sectional area (9). However, determining the most effective methods for measuring CPPe, CrCP, and RAP continues to pose a challenge. The evaluation of CrCP through linear regression analysis of pressure-flow data necessitates a sophisticated computational approach. In contrast, we employ three previously published methods for estimating CrCP that utilize the slope-intercept formula. These methods are more straightforward than the regression analysis of digital ABP and CBFV curves and can be readily applied to bedside assessments without the need to correct for time delays between ABP and CBFV.

This study aims to utilize clinical data from prior studies to conduct a secondary analysis, estimating CPPe, RAP, and CrCP of the intravascular common carotid artery using three different methods (10–12), and comparing these estimates with the reference method of linear regression.

## 2 Materials and methods

### 2.1 Research design, setting, and patients

The protocol was submitted to the hospital's ethics review committee prior to the trial. It was determined that no ethical approval was necessary, as the study did not interfere with or cause additional harm to the patients. All procedures were conducted in accordance with the Declaration of Helsinki, and all subjects provided informed consent.

This research involved 67 subjects who underwent cerebral digital subtraction angiography (DSA) due to cerebrovascular disease in the Department of Neurology between July 23, 2010, and February 9, 2011. The inclusion criteria were as follows: (1) patients exhibiting signs and symptoms of cerebrovascular disease; (2) CT and MRI examinations of the brain meeting the diagnostic criteria for acute ischemic stroke; and (3) patients who consented to undergo the DSA examination. Subjects were excluded if their condition could compromise the arterial blood pressure (ABP) survey in the common carotid artery and middle cerebral artery, such as severe stenosis or occlusion of blood vessels, or if poor middle cerebral artery (MCA) velocity signals were observed using transcranial Doppler (TCD) ultrasound. Specifically, the MCA velocity of four patients displayed a Doppler envelope difference. Consequently, 63 patients were included in the examination, while 44 were excluded due to cerebrovascular stenosis. The data from 19 patients were subsequently considered for secondary analysis.

### 2.2 Experimental procedures

All procedures were performed in an intervention room. All DSA examinations were performed using standard biplane fluoroscopy (Axiom Artis; Siemens Corporation, Munich, Germany). Since the operation was performed on the right side of patients, all monitoring indicators used were on the left side. Two investigators performed all measurements. The MCA velocity signal was acquired using transcranial Doppler ultrasound (TCD, Multidrop; DWL, Sipplingen, Germany) with a 2 MHz probe fixed on the head frame. All measurements used a temporal acoustic window and Doppler depth that yielded the maximum velocity. Non-invasive radial ABP was recorded from a radial artery with a continuous ABP monitor (CBM7000; Colin Medical Technology Corp., Komaki, Japan). After automatic calibration and correction, changes in blood pressure were continuously monitored with pulse fluctuations, and calibration was repeated once every 5 min.

All procedures were performed with the patient under local anesthesia and using a unilateral femoral approach. During the procedure, when the angiography catheter (451–503H5, Cordis Corp, Hialeah, Florida, USA) was 1–2 cm away from the opening of the innominate artery, we simultaneously monitored the aortic blood pressure, radial blood pressure, and MCA blood FV as a set of data. When the angiography catheter (451–514H0; Cordis Corp., Hialeah, FL, USA) was 2–3 cm from the CCA opening, we simultaneously monitored the common carotid ABP, radial ABP, and MCA blood FV as a set of data, after the three waveforms became stable. To mitigate the impact of heartbeat and respiratory fluctuations on the waveforms, Stability was defined as the rate of change per minute of less than 10%. The trend graph was plotted and recorded for 5 min, and all data were synchronized to the hard disk of the TCD machine.

### 2.3 Data analysis

**Figure 1:**
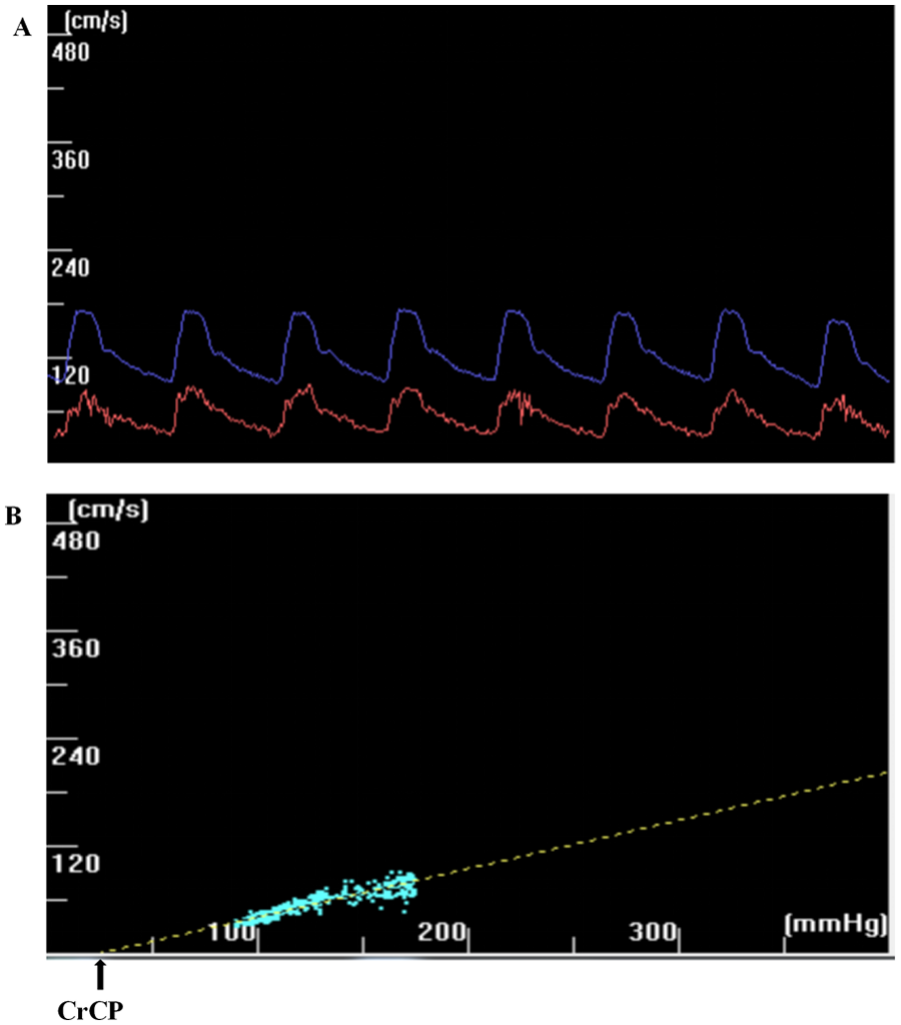
**(a)** The CBFV wave (red) and the ABP wave (blue) have been dragged horizontally, and the correlation coefficient between the two has reached the maximum. **(b)** The CrCP can be extrapolated through line regression (LR) of CBFV-ABP. The CrCP represents the intercept on the blood pressure axis (that is CBFV = 0).

We selected the ABP (abscissa) and Vmca (ordinate) curves corresponding to 8–10 continuous cardiac cycles (spanning at least one respiratory cycle) with envelope rules from raw data of previous studies. Customized software was used to move the blood FV wave and blood pressure wave relative to each other until the correlation coefficient between these two parameters reached a maximum. CrCP represents the blood pressure axis intercept (that is, CBFV = 0) (13) (Figure 1). The RAP is obtained from the inverse of the gradient of the regression line. The CrCP and RAP of each cardiac cycle were estimated by regression analysis of the pressure-velocity graphs. The mean values of CrCP and RAP for all heartbeats during these breathing cycles were used for further study. We calculated the systolic blood flow velocity (V_S_), diastolic blood flow velocity (V_S_), mean blood flow velocity (Vm), mean arterial pressure (MAP), systolic blood pressure (SAP), and diastolic blood pressure (DAP) values for each cardiac cycle. The other methods applied formulae to estimate CrCP.

Calculations:

Belfort et al. (14) $\mathrm{CrCP}(\mathrm{BEL})=\mathrm{MAP}-\mathrm{Vm}\times \frac{\left(\mathrm{MAP}-\mathrm{DBP}\right)}{\left(\mathrm{Vm}-\mathrm{Vd}\right)}$

Czosnyka et al. (12) $\mathrm{CrCP}(\mathrm{CZO})=\mathrm{SBP}-\mathrm{Vs}\times \frac{\left(\mathrm{SBP}-\mathrm{DBP}\right)}{\left(\mathrm{Vs}-\mathrm{Vd}\right)}$

Schmidt et al. (15) $\mathrm{CrCP}(\mathrm{SCH})=\mathrm{MAP}-(\mathrm{MAP}\times \frac{\mathrm{Vd}}{\mathrm{Vm}}+14)$

Subsequently, we calculated RAP as follows: RAP = (MAP−CrCP)/Vm. The CPPe for all methods was calculated as the difference between MAP and the respective CrCP.

### 2.4 Statistical analysis

Continuous classification and data are expressed as proportions (counts) and mean ± standard deviation. Paired Student's *t*-test was used to compare data between CCA. CPPe, CrCP, and RAP were compared [based on three methods: Belford, Czosnyka, and Schmidt (BEL, CZO, and SCH)] with the LR for analysis of variance (ANOVA). Further, multiple comparisons were conducted using Dunnett's method. The relationship and agreement were tested with Pearson or Spearman correlation tests and Bland–Altman analyses. Statistical analysis was performed using the IBM SPSS Statistics 25 software package (Armonk, NY: IBM Corp.) and GraphPad Prism version 7.0. *P* < 0.05 indicated statistical significance.

## 3 Results

The baseline characteristics of the nineteen patients are presented in Table 1. The mean age of the patients was 53.5 ± 11.6 years, with 73.7% being men. Additionally, the prevalence of a history of hypertension, diabetes, and cerebral infarction among the patients was 36.8%, 26.3%, and 15.8%, respectively.

**Table 1:** Baseline characteristics of 19 patients.

Variable	Mean + SD
Age (year)	53.5 ± 11.6
Male	14 (73.7%)
Temperature (^◦^C)	36.3 ± 0.3
Systolic blood pressure (mmHg)	142.1 ± 24.2
Diastolic blood pressure, (mmHg)	85.0 ± 14.7
Heart rate (beats/min)	71.19 ± 7.9
Hypertension	7 (36.8%)
Diabetes	5 (26.3%)
Hyperlipidemia	10 (15.9%)
Cerebral infarction	3 (15.8%)
Medications	
Beta-blockers	1 (5.3%)
Calcium channel blockers	4 (21.1%)
ACE inhibitors	2 (10.5%)
Smoking	9 (47.4%)
Drinking	1 (5.3%)

Values are mean + SD/N (%).

There were no significant differences observed between the right common carotid artery (RCCA) and the left common carotid artery (LCCA) in terms of vessel speed (Vs), diastolic velocity (Vd), mean velocity (Vm), mean arterial pressure (MAP), systolic arterial pressure (SAP), and diastolic arterial pressure (DAP) during the corresponding measurements. Additionally, no significant differences in cerebral perfusion pressure (CPPe), right atrial pressure (RAP), and cranial compliance pressure (CrCP) were detected using three different methods (Table 2).

**Table 2:** Comparison of parameters estimated by 4 different methods: between the common carotid artery.

	The difference between LCCA and RCCA		
	LCCA (*n* = 19)	RCCA (*n* = 19)	*P* value
VM (cm s^−1^)	56.3 ± 16.8	56.4 ± 15.6	0.782
VS (cm s^−1^)	90.5 ± 26.5	89.8 ± 25.3	0.525
VD (cm s^−1^)	33.5 ± 10.8	33.4 ± 10.4	0.843
MAP (mmHg)	106.9 ± 20.8	105.9 ± 20.6	0.182
SBP (mmHg)	155.3 ± 27.1	154.7 ± 26.6	0.755
DBP (mmHg)	83.2 ± 20.1	81.8 ± 19.6	0.147
CPP_LR_ (mmHg)	69.5 ± 19.9	70.6 ± 15.9	0.817
CPP_BEL_ (mmHg)	58.3 ± 13.8	58.5 ± 12.8	0.914
CPP_CZO_ (mmHg)	65.8 ± 16.9	66.5 ± 14.0	0.685
CPP_SCH_ (mmHg)	77.3 ± 13.7	76.4 ± 10.3	0.838
CrCP_LR_ (mmHg)	37.4 ± 26.0	35.3 ± 23.3	0.300
CrCP_BEL_ (mmHg)	48.7 ± 21.2	47.4 ± 18.1	0.497
CrCP_CZO_ (mmHg)	41.1 ± 22.9	39.4 ± 19.4	0.342
CrCP_SCH_ (mmHg)	29.7 ± 10.6	29.5 ± 24.7	0.961
RAP_LR_ (mmHg s cm^−1^)	0.78 ± 0.23	0.76 ± 0.21	0.778
RAP_BEL_ (mmHg s cm^−1^)	1.11 ± 0.38	1.11 ± 0.40	0.967
RAP_CZO_ (mmHg s cm^−1^)	1.24 ± 0.43	1.26 ± 0.43	0.715
RAP_SCH_ (mmHg s cm^−1^)	1.50 ± 0.55	1.47 ± 0.51	0.752

LCCA, left common carotid artery; RCCA, right common carotid artery; Vd, Vm, Vs = diastolic, mean, and systolic blood flow velocity; DAP, MAP, SAP, diastolic, mean, and systolic arterial pressure; CPP, cerebral perfusion pressure; CrCP, cerebral critical closing pressure; RAP, resistance–area product; LR (linear regression); Belford (BEL, 2-point: mean/diastolic); Czosnyka (CZO, 2-point: systolic/diastolic), and Schmidt (SCH, 2-point: systolic/diastolic). CPP_BEL_ indicates effective cerebral perfusion pressure based on BEL; CPP_CZO_ and CPP_SCH_, effective cerebral perfusion pressure based on CZO and SCH; CPP_LR_, effective cerebral perfusion pressure based on LR.

Compared to the reference method, the mean differences in CPPe and CrCP values for the left common carotid artery (LCCA) according to the SCH method were 7.7 (−5.0, 20.4) mmHg and −7.7 (−24.1, 8.6) mmHg, respectively. For the right common carotid artery (RCCA), the mean differences in CPPe and CrCP were 5.8 (−4.6, 16.2) mmHg and −5.8 (−22.6, 11.0) mmHg, respectively. According to the CZO calculation method, the mean differences in CPPe and CrCP values for LCCA were −3.7 (−16.4, 9.0) mmHg and 3.7 (−12.6, 20.0) mmHg, respectively. The average differences between CPPe and CrCP in RCCA were −4.1 (−14.5, 6.4) mmHg and 4.1 (−12.7, 20.9) mmHg, respectively. According to BEL's calculation method, the average differences in CPPe and CrCP values for LCCA were −11.2 (−23.9, 1.5) mmHg and 11.2 (−5.1, 27.6) mmHg, respectively. The mean differences between CPPe and CrCP in RCCA were −12.1 (−22.6, −1.7) mmHg and 12.2 (−4.6, 28.9) mmHg, respectively. It is noteworthy that RAP is not affected by this calculation method (Table 3).

**Table 3:** Differences between the 3 test methods of estimation (BEL, CZO, and SCH) and LR were tested by analysis of variance, with correction for multiple comparison (Dunnett).

	The difference between LCCA and RCCA			
	LCCA (*n* = 19)		RCCA (*n* = 19)	
	MD	*P* value	MD	*P* value
CPP_LR_ (mmHg)				–
CPP_BEL_ (mmHg)	−11.2 (−23.9, 1.5)	0.094	−12.1 (−22.6, −1.7)	0.018
CPP_CZO_ (mmHg)	−3.7 (−16.4, 9.0)	0.824	−4.1 (−14.5, 6.4)	0.668
CPP_SCH_ (mmHg)	7.7 (−5.0, 20.4)	0.332	5.8 (−4.6, 16.2)	0.406
CrCP_LR_ (mmHg)				–
CrCP_BEL_ (mmHg)	11.2 (−5.1, 27.6)	0.242	12.2 (−4.6, 28.9)	0.206
CrCP_CZO_ (mmHg)	3.7 (−12.6, 20.0)	0.906	4.1 (−12.7, 20.9)	0.887
CrCP_SCH_ (mmHg)	−7.7 (−24.1, 8.6)	0.532	−5.8 (−22.6, 11.0)	0.744
RAP_LR_ (mmHg s cm^−1^)				–
RAP_BEL_ (mmHg s cm^−1^)	0.33 (0.01, 0.65)	0.044	0.35 (0.04, 0.66)	0.024
RAP_CZO_ (mmHg s cm^−1^)	0.46 (0.14, 0.79)	0.003	0.50 (0.18, 0.81)	0.001
RAP_SCH_ (mmHg s cm^−1^)	0.72 (0.39, 1.04)	0.000	0.71 (0.39, 1.02)	0.000

LCCA, left common carotid artery; RCCA, right common carotid artery; CPP, cerebral perfusion pressure; CrCP, cerebral critical closing pressure; RAP, resistance–area product; LR (linear regression). Belford (BEL, 2-point: mean/diastolic), Czosnyka (CZO, 2-point: systolic/diastolic), and Schmidt (SCH, 2-point: systolic/diastolic). CPP_BEL_ indicates effective cerebral perfusion pressure based on BEL; CPP_CZO_ and CPP_SCH_, effective cerebral perfusion pressure based on CZO and SCH; CPP_LR_, effective cerebral perfusion pressure based on LR.

**Figure 2:**
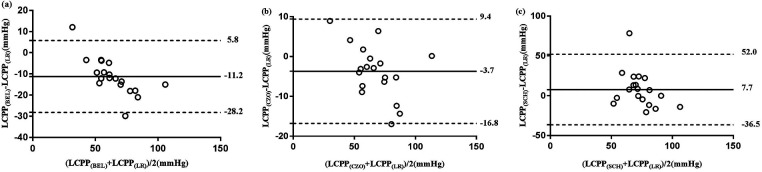
Bland-Altman analysis of the difference between the 3 test methods of CPPe estimation (BEL, CZO, and SCH) and reference method (LR). The solid horizontal line indicates the mean difference (bias) computed across the study population; dashed lines indicate the 95% CI limits for the mean, (**a**) mean differences of CPPe between BEL and LR, (**b**) mean differences of CPPe between CZO and LR, (**c**) mean differences of CPPe between SCH and LR.

The Bland–Altman analysis of CPPe and CrCP revealed a small mean bias when comparing CPP_CZO_ with CPP_LR_, Comparing CPP_LR_ measurements with CPP_BEL_, the mean bias was moderately higher with wider LoA (mean bias −11.2 mm Hg, LoA: −28.2 to 5.8 mm Hg). CPP_SCH_ showed less agreement with the reference method, with a mean bias of 7.7 mm Hg (wideranging LoA: −36.5 to 52.0 mm Hg). Biases and the 95% limits of agreement between RAP_LR_ values (reference method) and the RAP_BEL_, RAP_CZO_, and RAP_SCH_ calculation methods were small (Table 4; Figure 2).

**Table 4:** Bland–Altman analysis of the difference of CPPe, crCP and RAP between the 3 test methods of estimation (BEL, CZO, and SCH) and LR.

Dependent variable	Independent variable	Sample size	LCCA		RCCA	
			Mean difference	95% Limits of agreement	Mean difference	95% Limits of agreement
CPP_BEL_	CPP_LR_	19	−11.2	(−28.2 to 5.8)	12.2	(−5.5 to 29.8)
CPP_CZO_	CPP_LR_	19	−3.7	(−16.8 to 9.4)	4.1	(−10.3 to 18.5)
CPP_SCH_	CPP_LR_	19	7.7	(−36.5 to 52.0)	−5.8	(−46.5 to 34.9)
CrCP_BEL_	CrCP_LR_	19	11.2	(−5.8 to 28.2)	−12.1	(−29.8 to 5.5)
CrCP_CZO_	CrCP_LR_	19	3.7	(−9.4 to 16.8)	−4.1	(−18.5 to 10.3)
CrCP_SCH_	CrCP_LR_	19	−7.7	(−52.0 to 36.5)	5.8	(−34.9 to 46.5)
RAP_BEL_	RAP_LR_	19	0.33	(−0.83 to 1.49)	−0.35	(−1.51 to 0.81)
RAP_CZO_	RAP_LR_	19	0.46	(−0.79 to 1.72)	−0.50	(−1.72 to 0.72)
RAP_SCH_	RAP_LR_	19	0.72	(−0.59 to 2.02)	−0.71	(−2.00 to 0.59)

LCCA, left common carotid artery; RCCA, right common carotid artery; CPP, cerebral perfusion pressure; CrCP, cerebral critical closing pressure; RAP, resistance–area product; LR (linear regression). Belford (BEL, 2-point: mean/diastolic), Czosnyka (CZO, 2-point: systolic/diastolic), and Schmidt (SCH, 2-point: systolic/diastolic). CPPBEL indicates effective cerebral perfusion pressure based on BEL; CPP_CZO_ and CPP_SCH_, effective cerebral perfusion pressure based on CZO and SCH; CPP_LR_, effective cerebral perfusion pressure based on LR.

The Pearson correlation coefficients between the CPPe, CrCP, and RAP estimates, based on the BEL and CZO estimates as well as the LR, indicated a strong correlation. In contrast, the correlation between the CPPe and RAP assessed using the SCH estimations and the LR was slightly weaker (Table 5).

**Table 5:** Correlation of CPPe, crCP and RAP between the 3 test methods of estimation (BEL, CZO, and SCH) and LR.

Dependent variable	Independent variable	Sample size	LCCA		RCCA	
			Correlation coefficient	*P* value	Correlation coefficient	*P* value
CPP_BEL_	CPP_LR_	19	0.933	*P* < 0.0001	0.824	*P* < 0.0001
CPP_CZO_	CPP_LR_	19	0.948	*P* < 0.0001	0.886	*P* < 0.0001
CPP_SCH_	CPP_LR_	19	0.139	*P* = 0.570	−0.229	*P* = 0.346
CrCP_BEL_	CrCP_LR_	19	0.953	*P* < 0.0001	0.937	*P* < 0.0001
CrCP_CZO_	CrCP_LR_	19	0.971	*P* < 0.0001	0.958	*P* < 0.0001
CrCP_SCH_	CrCP_LR_	19	0.505	*P* < 0.05	0.628	*P* < 0.05
RAP_BEL_	RAP_LR_	19	−0.873	*P* < 0.0001	−0.872	*P* < 0.0001
RAP_CZO_	RAP_LR_	19	−0.916	*P* < 0.0001	−0.894	*P* < 0.0001
RAP_SCH_	RAP_LR_	19	−0.349	*P* = 0.143	−0.605	*P* < 0.05

LCCA, left common carotid artery; RCCA, right common carotid artery; CPP, cerebral perfusion pressure; CrCP, cerebral critical closing pressure; RAP, resistance–area product; LR (linear regression). Belford (BEL, 2-point: mean/diastolic), Czosnyka (CZO, 2-point: systolic/diastolic), and Schmidt (SCH, 2-point: systolic/diastolic). CPP_BEL_ indicates effective cerebral perfusion pressure based on BEL; CPP_CZO_ and CPP_SCH_, effective cerebral perfusion pressure based on CZO and SCH; CPP_LR_, effective cerebral perfusion pressure based on LR.

## 4 Discussion

In a secondary analysis of previously collected data, we validated three distinct estimates of CPPe, CrCP, and RAP in 19 patients undergoing digital subtraction cerebral angiography (DSA) for cerebrovascular disease. We employed custom software to align blood flow velocity (FV) waves with blood pressure waves, correcting for the time delay between arterial blood pressure (ABP) and cerebral blood flow velocity (CBFV) to minimize errors and enhance data accuracy. Our study demonstrated that the CPPe, CrCP, and RAP assessed from the common carotid artery (CCA) recordings validated the differences, relevance, and consistency among the three estimated test methods (BEL, CZO, and SCH) in comparison to the LR. The main findings of this paper are as follows: (1) the mean differences in the results of the CPPe and CrCP measurements based on the CZO and SCH calculation methods are minimal and align well with the LR; (2) when using LR as the reference method, the consistency of CPPe and CrCP was weak for the BEL method; and (3) RAP remains unaffected by these estimation methods.

Our data suggest that two-point extrapolation (CZO) estimates of CPPe and CrCP, derived from systolic and diastolic blood pressure data, are more accurate. This finding aligns with previous research indicating that, despite its limited capacity to predict CPP, the CZO estimation method effectively detects true CPP changes over time. Consequently, this method can serve as a non-invasive continuous monitoring tool for patients with mild to moderate head injuries (5, 12).

Our results indicate that the consistency of CPPe and CrCP obtained using the BEL method is weaker than that of the LR. This discrepancy may arise from the fact that the CrCP estimate calculated by the BEL method relies solely on the mean and diastolic period, omitting systolic values for both ABP and Vmca. Consequently, this approach reduces the distance of the velocity map, which could impact the accuracy of CrCP and RAP estimates. This omission may account for the broader BEL results observed in comparison to the standard approach (14).

Previous studies have demonstrated that, according to the formula proposed by Schmitt et al., there is a strong correlation between non-invasive CPPe and invasive CPPe values. The advantage of the SCH estimation method lies in its ability to reasonably detect genuine changes in cerebral perfusion pressure (CPP) over time. However, a notable disadvantage of this formula is the lack of a physiological or hemodynamic rationale; it is based solely on the investigator's 6 years of clinical experience. The authors derive the formula from the combination (multiplication) of the mean arterial blood pressure (ABP) and the nonlinear CPP-dependent factors FVd/FVm. When FVd is absent, the method exhibits a low saturation level of 14 mmHg (15). To the best of our knowledge, only a limited number of studies have employed SCH methods to evaluate CrCP, CPPe, or RAP. The CPPe values reported in previous studies utilizing the SCH method on average around 64 mmHg, which is lower than the findings of our study. This discrepancy may be attributed to the fact that those studies focused on patients with traumatic brain injury and ischemic heart disease, as elevated intracranial pressure and compromised cardiac function can significantly influence perfusion pressure (12, 15).

To our knowledge, there are few reports concerning the CPPe, CrCP, and RAP values measured by invasive CCA in patients undergoing brain DSA examinations. A previous study reported CPP values measured by invasive CCA in patients undergoing brain DSA, with their CPP values averaging approximately 110 mmHg. Notably, the CPPe values reported in that study were higher than those observed in our monitoring results. This discrepancy may be attributed to differences in the CPP evaluation methods employed in the earlier research, where CPP was calculated as CPP=CVAP−ICP. The formula used for calculating ICP was ICP=10.927×PI−1.284, and the measurement outcomes can also be influenced by variations in CCA measurement locations and monitoring equipment (16).

Comparing CrCP, CPPe, and RAP using pressure-flow velocity presents challenges due to variations in research design, objectives, statistical methods, and assessment techniques. Recently, Grüne validated alternative assessment methods based on the standard LR method, demonstrating good agreement (5). Furthermore, Giovanna evaluated three different equations to assess CPPe in 45 patients with severe traumatic brain injury (17). Their findings indicated that BEL estimation methods closely aligned with the actual measured CPPe. This similarity may be attributed to differences in the research subjects, invasive arterial blood pressure assessments, calculation methodologies, general anesthesia, and artificial ventilation.

In our study, the Cerebral Perfusion Pressure (CPP) and Cerebral perfusion pressure (CrCP) were obtained from the Common Carotid Artery (CCA). The mean CPP recorded from the CCA was 70 ± 17.9 mmHg, aligning with the Brain Trauma Foundation's current guidelines, which recommend a CPP of 60–70 mmHg (18). However, the CPP observed in our study was lower than that reported in previous research (19, 20). This discrepancy may be attributed to variations among subjects and the severity of their conditions.

The CrCP levels observed in this study were consistent with those reported in previous research involving populations with conditions such as head injury (21), cerebral vasospasm (8), cardiac surgery (22), and survivors of comatose cardiac arrest (23). However, earlier studies indicated lower CrCP values (24, 25), which may be attributed to their focus on healthy volunteers or to various physiological, pathological, or pharmacological states that could influence CrCP measurements (26). Additionally, the variability in blood pressure readings across different monitoring equipment may be due to the distinct data processing methods employed by various test systems. In contrast, RAP appears to be unaffected by its estimation method, likely because it is linked to more rapid myogenic responses associated with autoregulation, whereas CrCP reflects a slower metabolic response to cerebral blood flow (CBF) regulation (27, 28).

## 5 Conclusion

We validated three distinct methods for evaluating CPPe, CrCP, and RAP in a cohort of 19 patients with ischemic stroke. Our study demonstrates that the estimates of CPPe, CrCP, and RAP obtained using CZO are simpler and more user-friendly for bedside assessment compared to standard methods.

### 5.1 Limitations

This study has several limitations. First, the findings are applicable only to patients with access to intravascular blood pressure monitoring in the common carotid artery (CCA). Second, autoregulation is progressively diminished in cases where cerebral perfusion pressure (CPP) falls below 40 mmHg and in the presence of severe vascular stenosis. Consequently, the pressure-flow graph may exhibit nonlinearity. Most methods for estimating cerebrovascular resistance (CrCP) are constrained to the linear range of the pressure-flow relationship, as they are derived from linear pressure-flow (velocity) graph analyses, yielding only “hypothetical” or “apparent” values. Thus, LR as the reference method in the classification of CrCP remains a subject of debate. Future investigations should encompass diverse patient populations, ideally including those with varying degrees of cerebrovascular stenosis who are undergoing interventional therapy.

## Data Availability

The original contributions presented in the study are included in the article/Supplementary Material, further inquiries can be directed to the corresponding authors.
